# Interspecific competition models and resource inequality between individuals

**DOI:** 10.1098/rsos.240501

**Published:** 2024-08-14

**Authors:** Masahiro Anazawa

**Affiliations:** ^1^ Department of Applied Chemistry and Environment, Tohoku Institute of Technology, Sendai 982-8577, Japan

**Keywords:** Hassell–Comins model, first-principles derivation, individual, contest competition, scramble competition

## Abstract

Most classical discrete-time population models of interspecific competition have emerged as population-level phenomenological models with no evident basis at the individual level. This study shows that the Hassell–Comins model, a widely used discrete-time model of interspecific competition, can be derived in a bottom-up manner from a simple model of random resource competition between individuals of two species as an expression of expected population sizes in the next generation. The random competition leads to inequalities in resource allocation between individuals, which are related to the key parameters of the Hassell–Comins model that determine the density dependence of each species. The relationship between population-level parameters, such as intra- and interspecific competition coefficients, and individual-level parameters is discussed in detail, as is how the derived competition equations depend on the degree of inequality within each species. By considering limits of maximum or minimum resource inequality within each species, the derived model can describe interspecific competition for various combinations of two species exhibiting ideal scramble or ideal contest intraspecific competition.

## 1. Introduction

Discrete-time population models $X_{t+1}=f(X_{t})$ , which express the population size at one generation $X_{t+1}$ as a function of the population size at the previous generation $X_{t}$ , are often used to describe the population dynamics of seasonally reproducing species, such as insects. Well-known examples include the Ricker model [1], Beverton–Holt model [2] and Hassell model [3]. Most classical discrete-time models were initially introduced as phenomenological models at the population level, rather than being derived based on interactions between individuals. However, there have been recent advances in research that are aimed at deriving these models from first principles. Given that they were originally phenomenological, classical discrete-time models do not always have to be derived from fundamental processes. However, if a phenomenological model can be derived from individual-level processes, a deeper understanding of the model would be gained, e.g. suggesting a relationship between phenomenological parameters and individual-level processes. This study focuses on the first-principles derivation of discrete-time models of interspecific competition, an area that has received scant attention.

The Hassell–Comins model is a commonly used model of interspecific competition and is given by [4]:


(1.1*a*) $$X_{t+1}=\frac{\lambda_{1}\;X_{t}}{{\left[1+a_{1}\left(X_{t}+b_{12}Y_{t}\right)\right]}^{\theta_{1}}},$$

(1.1*b*) $$\begin{array}{rl} Y_{t+1} & =\frac{\lambda_{2}Y_{t}}{[1+a_{2}(Y_{t}+b_{21}X_{t}){]}^{\theta_{2}}}, \end{array}$$

where $X_{t}$ and $Y_{t}$ are the population sizes of two species at generation t . This model can describe different density dependencies by changing its exponents $\theta_{1}$ and $\theta_{2}$ , and it has been widely used owing to this flexibility, for example to analyse data on interspecific competition [5–10]. In general, interspecific competition models are often introduced through intuitive modification of single-species population models. That is, if the original population model is $X_{t+1}=X_{t}\;g(X_{t})$ , $X_{t}$ in $g(X_{t})$ is replaced by $X_{t}+b_{12}Y_{t}$ , where $b_{12}$ is a phenomenological interspecific competition coefficient. The Hassell–Comins model was introduced in the same fashion from the Hassell model [3,11]


(1.2) $$X_{t+1}=\frac{\lambda X_{t}}{(1+aX_{t}{)}^{\theta }}.$$

**Figure 1.**
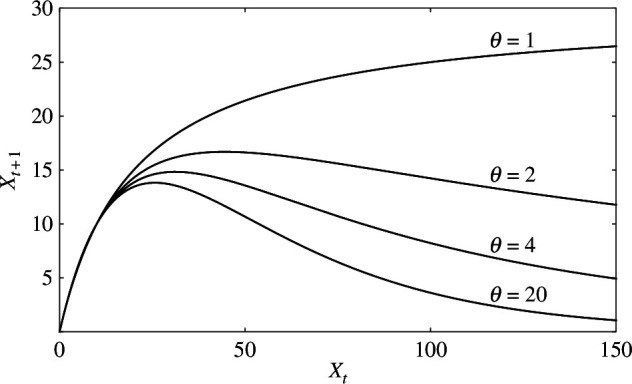
Reproduction curves of the Hassell model $X_{t+1}=\lambda X_{t}/(1+aX_{t}{)}^{\theta }$ for different values of exponent θ . λ=1.5 ; a is determined by the condition $X_{*}=10$ .

Although such procedures are commonly used, they are only intuitive methods and their rationale is unclear, for example the circumstances under which they can be justified. Few studies have addressed their justification from first principles based on individual-level processes. The Hassell model (1.2) shows varying density dependencies by adjusting the exponent θ (figure 1). When θ=1 , the reproduction curve increases monotonically towards a constant, showing an exact compensating density dependence for large population sizes. When θ>1 , the curve rises to a maximum and then falls, showing an overcompensating density dependence, the degree of which increases with θ . Hassell interpreted that θ=1 corresponds to ideal contest competition, and the limit θ→∞ corresponds to ideal scramble competition [3]. Here, contest and scramble are the two types of intraspecific competition for resources introduced by Nicholson [12]. In ideal contest competition, a few individuals monopolize the resources, resulting in the next generation’s population size being largely independent of the current large population size. In ideal scramble, resources are almost equally distributed, and consequently, the probability of reproduction falls rapidly as population size increases. These two types of competition are related to inequality in resource distribution between individuals [13,14]. In ideal contest, the inequality between individuals is large, whereas in ideal scramble, it is small. Therefore, the exponent θ of the Hassell model should be related to the inequality. For the Hassell–Comins model, the situation is complicated because the two species have separate exponents $\theta_{1}$ and $\theta_{2}$ , but they should still be related to inequalities between individuals.

The Hassell model is derived from first principles through multiple approaches. They can be broadly classified into three approaches. In the first approach, the Hassell model is derived from a continuous-time model describing dynamics within a year as a model describing dynamics between years [15–20]. The second approach derives discrete-time models in situations where individuals in a population are distributed across many resource sites or patches (site-based frameworks). In this approach, many population models, including the Hassell model, are derived by varying individual interactions at each site and individual distributions across patches [21–25]. Several interspecific competition models, including the Hassell–Comins model, can also be derived in the same approach [26]. While both approaches derive Hassell models, their exponents are unrelated to inequality in resource allocation between individuals. Therefore, these derivations do not reflect the scenario assumed by the original Hassell model [3]. This problem is solved by the third approach [27], which derives a Hassell model with an exponent related to such inequality as an expression of the expected population size in the next generation from random resource competition between individuals. This derivation is characterized by assuming that each individual can obtain only a fixed amount of resources (resource unit) at a time. This idealized assumption is made in order to examine the effect of resource inequality in as simple a setting as possible. When resources are randomly distributed to individuals under this assumption, the degree of inequality in the resource distribution depends on the size of the resource unit, and the derived Hassell model comprises an exponent related to this inequality. If the same approach can be applied to competition between individuals of two species, it might be possible to derive a Hassell–Comins model with exponents related to inequality in resource allocation. The extension to two species may seem straightforward, but different species can have different resource unit sizes. This fact precludes a straightforward extension of the single-species method to two species. This approach has not yet been extended to two species.

This study aims to extend the derivation of the Hassell model using the third approach [27] to two species and shows that from a simple model of random resource competition between individuals of two species, the expected population sizes in the next generation are described by the same functions as in the Hassell–Comins model. It also examines how phenomenological parameters, such as intra- and interspecific competition coefficients in the derived Hassell–Comins model, are related to individual-level parameters. In addition, it examines how the derived model depends on inequality within each species and considers competition models for specific combinations of competition types, such as ideal contest versus ideal scramble.

## 2. Derivation of discrete-time models

### 2.1. Hassell model

The derivation of the Hassell model in Anazawa [27], on which this study is based, is first outlined. Suppose that N individuals in a population compete for a total amount R of resources. Each individual is assumed to obtain only a fixed amount u of resources at a time. This situation is practically the same as if the resources were divided into many chunks of this fixed size (resource units), which are collected randomly by individuals. The coefficient of variation (CV) for the amount of resources obtained by an individual is inversely proportional to the total number of resource units M , the integer part of R/u . Thus, increasing u while holding R constant increases the inequality in resource allocation between individuals. Each individual is assumed to require at least s resource units to reproduce. If the value of R is unknown and only its probability distribution is known to be exponential with mean $\bar{R}$ , the expected population size at the next generation is calculated to be


(2.1) $$f(N)=\frac{\lambda N}{{\left[1+(e^{u/\bar{R}}-1)N\right]}^{s}}\;,$$

where λ is the expected number of offspring produced by a reproductive individual. The exponent of the Hassell model derived here is s , the number of resource units required for reproduction. Given that u is related to the degree of inequality between individuals and s=w/u , where w is the actual resource amount required for reproduction, this exponent is indeed related to the inequality. The inequality is greatest when u=w , that is when s=1 , and approaches 0 as s increases, maintaining w=us constant.

The exponential distribution of R assumed here was interpreted in terms of the principle of maximum entropy [28,29]. In general, if only the expected value of a continuous random variable is known, without any other knowledge of its probability distribution, the distribution that maximizes information entropy is the exponential distribution. Since such a distribution is the least biased distribution consistent with our prior knowledge, the Hassell model (2.1) can be interpreted as the most natural estimate of the population size at the next generation when we only know the expected value, not the actual value, of the total resource amount. Another possible interpretation of the R distribution is that it represents random variations of R over time. If the time series of R follows the exponential distribution, the average relationship between population sizes in two consecutive generations can be described by the Hassell model (2.1).

### 2.2. Interspecific competition model (simple case)

Next, we extend the above derivation of the Hassell model to two species. Two species can have different resource unit sizes. However, this assumption considerably complicates the derivation of a competition model, so that this subsection first considers the case where both species have the same resource unit size (the general case is addressed in §2.3). In this case, the ideas for the single-species case can be extended almost straightforwardly to the two-species case.

**Figure 2.**
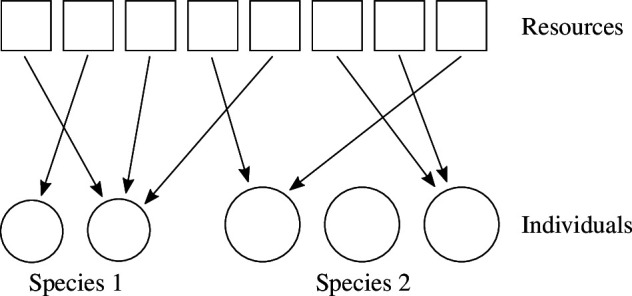
Competition for limited resources between individuals of two species for the simple case in §2.2. If any individual of both species can obtain only a fixed amount of resources (resource unit) at a time, the resources are essentially the same as being divided into many chunks of this constant size, which are randomly distributed among the individuals.


Suppose that $N_{1}$ individuals of species 1 and $N_{2}$ individuals of species 2 compete for a total amount R of resources. If any individual can obtain only u of resources at a time, there are effectively M resource units in total (the integer part of R/u ), which are randomly distributed among the individuals (figure 2). If the two species have different probabilities of an individual obtaining a resource unit, in the ratio of $\alpha_{1}:\alpha_{2}$ , then the probability of a species 1 individual obtaining exactly $m_{1}$ ( ≤M ) resource units is:

(2.2) p1(m1;M)=ρ1m1(1−ρ1)M−m1(Mm1),

where


(2.3) $$\rho_{1}=\frac{\alpha_{1}}{\alpha_{1}N_{1}+\alpha_{2}N_{2}}=\frac{1}{N_{1}+\frac{\alpha_{2}}{\alpha_{1}}N_{2}}.$$

If a species 1 individual requires at least $s_{1}$ resource units to reproduce, the expected population size of species 1 at the next generation is written as:


$$\begin{array}{r} f_{1}(N_{1},N_{2};M)=\lambda_{1}N_{1}\sum_{m_{1}=s_{1}}^{M}p_{1}(m_{1};M), \end{array}$$

where $\lambda_{1}$ is the expected number of offspring produced by a reproductive individual of species 1.

Assume that the actual value of R is unknown and its probability density follows an exponential distribution:


(2.4) $$q(R)=e^{-R/\bar{R}}/\bar{R},$$

where $\bar{R}$ is the expected value of R , then the probability of having a total of M resource units, $P(M;\bar{R})$ , follows a geometric distribution:


(2.5) $$\begin{array}{ccc} & P(M;\bar{R})=(1-e^{-u/\bar{R}}\;)\;e^{-Mu/\bar{R}}. & \end{array}$$

With this distribution, the expected population size of species 1 at the next generation can be written as:


$$f_{1}(N_{1},N_{2})=\lambda_{1}N_{1}\sum_{M=0}^{\infty }\sum_{m_{1}=s_{1}}^{\infty }p_{1}(m_{1};M)\;P(M;\bar{R}),$$

where $p_{1}(m_{1};M)=0$ for $m_{1}>M$ . By interchanging the order of the two summations above, this equation can be written as:


(2.6) $$f_{1}(N_{1},N_{2})=\lambda_{1}N_{1}\sum_{m_{1}=s_{1}}^{\infty }{\hat{p}}_{1}(m_{1};\bar{R}),$$

where


(2.7) $${\hat{p}}_{1}(m_{1};\bar{R})=\sum_{M=0}^{\infty }p_{1}(m_{1};M)\;P(M;\bar{R})$$

represents the probability distribution of $m_{1}$ when R follows the exponential distribution. Calculating the sum in equation (2.7), then substituting the result into the right-hand side of equation (2.6), and obtaining the sum gives the expected population size of species 1 at the next generation (see appendix A for details). The calculations for species 2 are similar. Finally, the expected population sizes of the two species at the next generation are expressed as:


(2.8*a*) $$\begin{array}{ll} f_{1}(N_{1},N_{2}) & =\lambda_{1}N_{1}{\left[1+(e^{u/\bar{R}}-1)(N_{1}+\frac{\alpha_{2}}{\alpha_{1}}N_{2})\right]}^{-s_{1}}, \end{array}$$

(2.8*b*) $$\begin{array}{ll} f_{2}(N_{1},N_{2}) & =\lambda_{2}N_{2}{\left[1+(e^{u/\bar{R}}-1)(N_{2}+\frac{\alpha_{1}}{\alpha_{2}}N_{1})\right]}^{-s_{2}}, \end{array}$$

where $\lambda_{2}$ and $s_{2}$ are the same as species 1. These functional forms are straightforward extensions of equation (2.1), consistent with the Hassell–Comins model (1.1). As the two species have the same resource unit size, the inequalities within each species cannot be changed independently in this model.

### 2.3. Interspecific competition model (general case)

**Figure 3.**
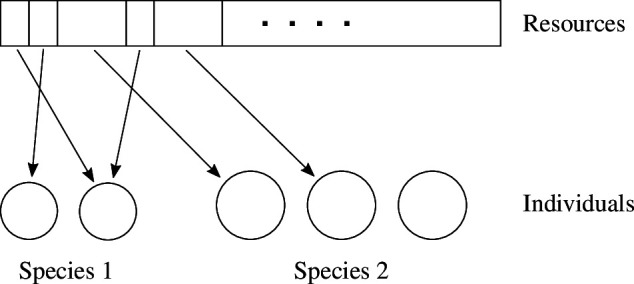
Competition between individuals of two species for the general case in §2.3. A random selection of an individual and its acquisition of a resource unit is repeated until the resources are exhausted. If the two species have different resource unit sizes, it cannot be assumed that the resources are pre-divided into many fragments of the same size.


Consider the general case where two species have different resource unit sizes $u_{1}$ and $u_{2}$ . Here, the total number of resource units M cannot be uniquely determined in advance (figure 3). This fact considerably complicates the derivation process. Presenting the result first, we can obtain the following expressions for the expected population sizes of the two species at the next generation:


(2.9*a*) $$\begin{array}{ll} f_{1}(N_{1},N_{2}) & =\lambda_{1}N_{1}{\left[1+(e^{u_{1}/\bar{R}}-1)(N_{1}+b_{12}N_{2})\right]}^{-s_{1}}, \end{array}$$

(2.9*b*) $$\begin{array}{ll} f_{2}(N_{1},N_{2}) & =\lambda_{2}N_{2}{\left[1+(e^{u_{2}/\bar{R}}-1)(N_{2}+b_{21}N_{1})\right]}^{-s_{2}}, \end{array}$$

where


(2.10) $$b_{12}=\frac{1-e^{-u_{2}/\bar{R}}}{1-e^{-u_{1}/\bar{R}}}\;\frac{\alpha_{2}}{\alpha_{1}},\;b_{21}=\frac{1-e^{-u_{1}/\bar{R}}}{1-e^{-u_{2}/\bar{R}}}\;\frac{\alpha_{1}}{\alpha_{2}},$$

are the interspecific competition coefficients expressed as a function of individual-level parameters. When $u_{1}=u_{2}=u$ , equation (2.9) reduces to equation (2.8). When $u_{i}\ll \bar{R}$ , by Taylor-expanding $e^{\pm u_{i}/\bar{R}}$ to the first order of $u_{i}/\bar{R}$ , equation (2.9) become a simple model:


$$\begin{array}{rl} f_{1}(N_{1},N_{2}) & =\lambda_{1}N_{1}{\left[1+\frac{u_{1}}{\bar{R}}(N_{1}+\frac{u_{2}}{u_{1}}\frac{\alpha_{2}}{\alpha_{1}}N_{2})\right]}^{-s_{1}},\\ f_{2}(N_{1},N_{2}) & =\lambda_{2}N_{2}{\left[1+\frac{u_{2}}{\bar{R}}(N_{2}+\frac{u_{1}}{u_{2}}\frac{\alpha_{1}}{\alpha_{2}}N_{1})\right]}^{-s_{2}}. \end{array}$$

The interpretation of the result is discussed in the next section, and the main ideas behind the derivation of (equation (2.9)) is outlined below (see appendix B for details).

Assume that there is an amount R of resources initially and randomly select an individual to allocate a resource unit $u_{i}$ specific to its species repeatedly (see figure 3). The probability of an individual of two species being selected is $\rho_{1}$ in equation (2.3) and $\rho_{2}=1-\rho_{1}$ , respectively, as in §2.2. If the remaining resources are less than the resource unit size of the selected individual, the individual receives all remaining resources. After resources are exhausted, only those individuals that have acquired $u_{i}s_{i}$ or more resources for each species i reproduce, and a reproducing individual produces, on average, $\lambda_{i}$ offspring. Let $M_{1}$ and $M_{2}$ be the total number of resource units allocated to each species until the resources are exhausted. However, the last allocated resources are not included in the counts of $M_{1}$ and $M_{2}$ as they are less than $u_{i}$ and do not affect the probability of reproduction. To calculate the expected population sizes at the next generation, we must first determine the probability $P(M_{1},M_{2};R)$ that the allocation $\left(M_{1},M_{2}\right)$ will be realized when R is given. Let us follow the resource allocation process in detail. Each time a randomly selected individual acquires $u_{i}$ of resources, the remaining resources decrease, and eventually become less than $u_{1}$ or $u_{2}$ . These remaining resources are all consumed by the last selected individual and the resource allocation process ends. In general, resource allocation ends when: (i) a species 1 individual receives all remaining resources less than $u_{1}$ , or when (ii) a species 2 individual receives all remaining resources less than $u_{2}$ . For the allocation $\left(M_{1},M_{2}\right)$ to be realized by (i) or (ii), the total resource R must satisfy the following inequalities, respectively,


$$\begin{array}{rl} & u_{1}M_{1}+u_{2}M_{2}\leq R<u_{1}(M_{1}+1)+u_{2}M_{2},\\ & u_{1}M_{1}+u_{2}M_{2}\leq R<u_{1}M_{1}+u_{2}(M_{2}+1). \end{array}$$

Considering these conditions, the allocation $\left(M_{1},M_{2}\right)$ will be realized with the probability:


(2.11) P(M1,M2;R)=(M1+M2M1)q1M1q2M2⋅[q1I(u1M1+u2M2≤R<u1(M1+1)+u2M2)+q2I(u1M1+u2M2≤R<u1M1+u2(M2+1))],

where I(condition) represents 1 if the condition is satisfied and 0 otherwise, and


$$q_{1}=\frac{\alpha_{1}N_{1}}{\alpha_{1}N_{1}+\alpha_{2}N_{2}},\;q_{2}=\frac{\alpha_{2}N_{2}}{\alpha_{1}N_{1}+\alpha_{2}N_{2}}.$$

The allocation of resources less than a resource unit is not involved in the combination factor on the right-hand side of equation (2.11). This is because it must be performed last, even though the other resource allocations can be rearranged. When *R* follows the exponential distribution (2.4), combining equation (2.11) and


$$Prob[A\leq R<B]=e^{-A/\bar{R}}-e^{-B/\bar{R}}$$

shows that the allocation $\left(M_{1},M_{2}\right)$ can be realized with the probability:


(2.12) P(M1,M2;R¯)=(M1+M2M1)(e−u1/R¯q1)M1(e−u2/R¯q2)M2⋅(1−e−u1/R¯q1−e−u2/R¯q2).

This distribution can be confirmed to be normalized to 1. Summing this distribution over all values of $M_{2}$ leads to the marginal distribution of $M_{1}$ ,


(2.13) $$P_{1}(M_{1};\bar{R})=\sum_{M_{2}=0}^{\infty }P(M_{1},M_{2};\bar{R}),$$

which results in a geometric distribution, whose mean is


$${\bar{M}}_{1}=N_{1}[\;(1-e^{-u_{1}/\bar{R}})(N_{1}+b_{12}N_{2})\;{]}^{-1}.$$

The calculations that follow are similar to those in §2.2. If $M_{1}$ follows the distribution resulting from (2.13), the probability of a species 1 individual acquiring exactly $m_{1}$ resource units, ${\hat{p}}_{1}(m_{1};\bar{R})$ , can be determined as follows:


(2.14) $${\hat{p}}_{1}(m_{1};\bar{R})=\sum_{M_{1}=0}^{\infty }p_{1}(m_{1};M_{1})\;P_{1}(M_{1};\bar{R}),$$

where $p_{1}(m_{1};M_{1})$ is the distribution of $m_{1}$ when $M_{1}$ is fixed,


p1(m1;M1)=(1N1)m1(1−1N1)M1−m1(M1m1)

for $m_{1}\leq M_{1}$ , and $p_{1}(m_{1};M_{1})=0$ for $m_{1}>M_{1}$ . Calculating the sum on the right-hand side of equation (2.14) yields a geometric distribution with mean


(2.15) $${\bar{m}}_{1}=[\;(1-e^{-u_{1}/\bar{R}})(N_{1}+b_{12}N_{2})\;{]}^{-1}.$$

With the distribution ${\hat{p}}_{1}(m_{1};\bar{R})$ obtained here, the expected population size of species 1 at the next generation can be written as:


$$f_{1}(N_{1},N_{2})=\lambda_{1}N_{1}\sum_{m_{1}=s_{1}}^{\infty }{\hat{p}}_{1}(m_{1};\bar{R}).$$

Calculating the sum on the right-hand side of this equation leads equation (2.9*a*). Equation (2.9*b*) can be derived similarly.

## 3. Interpretation of the derived models

### 3.1. Competition coefficients

The derived model (2.9) has the intraspecific $a_{i}=e^{u_{i}/\bar{R}}-1$ and interspecific $b_{ij}$ competition coefficients. These phenomenological coefficients at the population level are expressed as functions of more fundamental parameters such as $\bar{R}$ , $u_{i}$ , $u_{j}$ , $\alpha_{i}$ and $\alpha_{j}$ . These coefficients are the results of the calculations in the previous section, but is it possible to intuitively understand why they have such functional forms? In the following, $a_{1}$ and $b_{12}$ are considered.

Let $M_{i\;\max}$ be the maximum number of resource units of species i that can be obtained from resource R (the integer part of $R/u_{i}$ ). Note that when R follows the exponential distribution, the expected value of $M_{i\;\max}$ is


(3.1) $${\bar{M}}_{i\;max}=(e^{u_{i}/\bar{R}}-1{)}^{-1}.$$

This shows that the intraspecific competition coefficient $a_{1}$ is equal to $1/{\bar{M}}_{1\;\max}$ . This is intuitively convincing because it is natural that higher values of ${\bar{M}}_{1\;\max}$ lead to lower effects of intraspecific competition.

The interspecific competition coefficient $b_{12}$ should represent the competitive effect of a species 2 individual on species 1 compared with that of a species 1 individual. As $1-e^{-u_{i}/\bar{R}}=(e^{u_{i}/\bar{R}}-1)e^{-u_{i}/\bar{R}}$ , $b_{12}$ in equation (2.10) can be written as:


(3.2) $$b_{12}=\frac{{\bar{M}}_{1\;max}}{{\bar{M}}_{2\;max}}\cdot \frac{e^{-u_{2}/\bar{R}}}{e^{-u_{1}/\bar{R}}}\frac{\alpha_{2}}{\alpha_{1}}.$$

As the competitive effect of a species i individual should be proportional to $1/{\bar{M}}_{i\;\max}$ , the inclusion of ${\bar{M}}_{1\;\max}/{\bar{M}}_{2\;\max}$ in $b_{12}$ is valid, but the following factor is included:


(3.3) $$c_{12}=\frac{e^{-u_{2}/\bar{R}}}{e^{-u_{1}/\bar{R}}}\frac{\alpha_{2}}{\alpha_{1}}.$$

What does this factor imply? First, note that $e^{-u_{i}/\bar{R}}$ equals the probability that the remaining resource is $u_{i}$ or more at any given point in the resource allocation process. Specifically, when $R^{\prime \prime }$ of the total resource R is already allocated, the probability that the remaining resource $R^{\prime }=R-R^{\prime \prime }$ is $u_{i}$ or greater is


$$Prob[R^{\prime }\geq u_{i}]=\frac{Prob[R\geq R^{\prime \prime }+u_{i}]}{Prob[R\geq R^{\prime \prime }]}=e^{-u_{i}/\bar{R}},$$

which is independent of $R^{\prime \prime }$ owing to the exponential distribution. $R^{\prime }\geq u_{i}$ must be satisfied for a species i individual, if selected the next time, to acquire $u_{i}$ of resources. Given the above equation, equation (3.3) can be written as:


$$c_{12}=\frac{Prob[R^{\prime }\geq u_{2}]\alpha_{2}}{Prob[R^{\prime }\geq u_{1}]\alpha_{1}},$$

which represents the ratio of the probability of a species 2 individual acquiring $u_{2}$ to the probability of a species 1 individual acquiring $u_{1}$ . From this viewpoint, it is natural that $b_{12}$ includes the factor $c_{12}$ , and equation (3.2) is intuitively satisfactory.

### 3.2. Effects of resource inequality

A change in $u_{i}$ is expected to change the inequality in resource allocation between individuals of species i . We examine how the inequality within species i varies with $u_{i}$ and how the competition model (2.9) changes accordingly (the case of species 1 considered). For that, we first need an index of the degree of inequality within species 1. When the total number of resource units allocated to species 1, $M_{1}$ , is fixed, the squared coefficient of variation of the number of resource units $m_{1}$ acquired by a species 1 individual is given by:


(3.4) $$({CV}_{1}{)}^{2}=\frac{N_{1}}{M_{1}}\left(1-\frac{1}{N_{1}}\right).$$

As $M_{1}$ is not a constant, this equation needs to be averaged according to the distribution (2.13) of $M_{1}$ . However, as $M_{1}$ on the right-hand side can be 0 , it is not possible to calculate the average. To avoid this difficulty, the inverse of equation (3.4) is averaged and the inverse of the result is used as an index of the inequality within species 1. This procedure is equivalent to replacing $M_{1}$ in equation (3.4) by ${\bar{M}}_{1}$ , giving the inequality index:


(3.5) $$\eta_{1}=(e^{u_{1}/\bar{R}}-1)(N_{1}+b_{12}N_{2})\left(1-\frac{1}{N_{1}}\right).$$

Using this index, we consider how a change in $u_{1}$ changes the inequality within species 1, and how equation (2.9*a*) changes accordingly. To examine the pure effect of changing the inequality within species 1, conditions other than the inequality must be held constant as $u_{1}$ is varied. It is appropriate to maintain ${\bar{m}}_{1}/{\bar{M}}_{1\;\max}$ and $s_{1}/{\bar{M}}_{1\;\max}$ constant. For $u_{i}\ll \bar{R}$ , these quantities are equal to ${\bar{m}}_{1}u_{1}/\bar{R}$ and $s_{1}u_{1}/\bar{R}$ , respectively. Thus, holding them constant is equivalent to fixing the expected actual resource acquired by a species 1 individual and the actual resource required for reproduction. From equations (2.15) and (3.1), we have:


(3.6) $${\bar{m}}_{1}/{\bar{M}}_{1\;max}=(N_{1}+b_{12}N_{2}{)}^{-1},$$

(3.7) $$s_{1}/{\bar{M}}_{1\;max}=s_{1}(e^{u_{1}/\bar{R}}-1).$$

To maintain the first quantity constant when $N_{1}$ and $N_{2}$ are fixed, $b_{12}$ should be maintained constant. This is possible by varying $\alpha_{2}/\alpha_{1}$ simultaneously with $u_{1}$ , as observed from equation (2.10). If equation (3.7) is set to a constant $1/k_{1}$ , $\eta_{1}$ can be written as:


$$\eta_{1}=\frac{1}{s_{1}k_{1}}(N_{1}+b_{12}N_{2})\left(1-\frac{1}{N_{1}}\right),$$

where $k_{1}$ is interpreted as the expected maximum number of species 1 individuals that can reproduce. This index is inversely proportional to $s_{1}$ , with a maximum at $s_{1}=1$ , decreasing as $s_{1}$ increases, if $N_{1}$ and $N_{2}$ are fixed.

Under the aforementioned conditions, equation (2.9*a*) becomes:


(3.8) $$f_{1}(N_{1},N_{2})=\lambda_{1}N_{1}{\left[1+\frac{1}{s_{1}k_{1}}(N_{1}+b_{12}N_{2})\right]}^{-s_{1}}.$$

**Figure 4.**
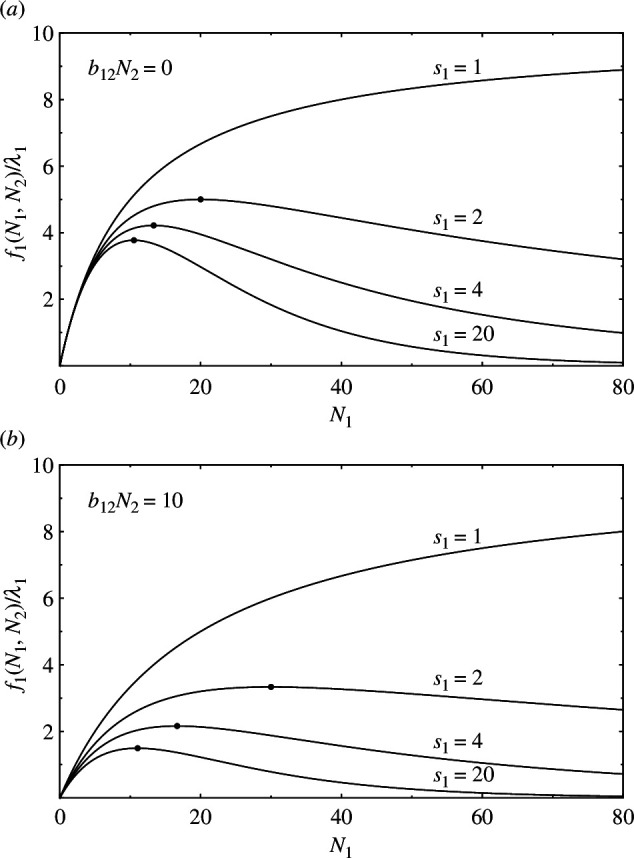
Reproduction curves of species 1, $f_{1}(N_{1},N_{2})$ from equation (3.8), as a function of $N_{1}$ ( $k_{1}=10$ ): (*a*) $b_{12}N_{2}=0$ and (*b*) $b_{12}N_{2}=10$ . For small $N_{1}$ , $f_{1}$ shows no dependence on $s_{1}$ , that is no dependence on the level of inequality within species 1. As $N_{1}$ increases, $f_{1}$ begins to show differences owing to $s_{1}$ . For $s_{1}\geq 2$ , $f_{1}$ has a maximum point (indicated by the dots).


Figure 4 shows $f_{1}/\lambda_{1}$ as a function of $N_{1}$ with $N_{2}$ fixed ( $k_{1}=10$ ). Note that when $(N_{1}+b_{12}N_{2})/k_{1}≃0$ , equation (3.8) can be written from its Taylor expansion as:

$$f_{1}(N_{1},N_{2})≃\lambda_{1}N_{1}\left[1-\frac{1}{k_{1}}(N_{1}+b_{12}N_{2})\right],$$

which is independent of $s_{1}$ . Thus, when $(N_{1}+b_{12}N_{2})/k_{1}$ is small, $f_{1}$ shows no dependence on $s_{1}$ . As $(N_{1}+b_{12}N_{2})/k_{1}$ increases, it begins to show differences owing to $s_{1}$ . As shown in figure 4, for $s_{1}=1$ , $f_{1}$ asymptotically approaches $\lambda_{1}k_{1}$ with $N_{1}$ . For $s_{1}\geq 2$ , $f_{1}$ has a maximum, and both its value and the value of $N_{1}$ at the maximum point decrease as $s_{1}$ increases. In the limit $s_{1}\to \infty$ , $f_{1}$ has a maximum of $\lambda_{1}k_{1}\exp[-(k_{1}+b_{12}N_{2})/k_{1}]$ at $N_{1}=k_{1}$ . The larger the $s_{1}$ value, the faster $f_{1}$ decreases after reaching its peak. This is because a larger value of $s_{1}$ results in smaller differences in acquired resources between individuals, and thus a more abrupt increase in non-reproducing individuals as individuals increase. As $f_{1}$ is independent of $s_{2}$ , the inequality within species 2 does not affect the probability of reproduction of species 1.

### 3.3. Extreme case models

Now, consider the competition model (2.9) with maximum or minimum inequality within each species. First, the competition model with maximum inequalities for both species is given by equation (2.9) with $s_{1}=s_{2}=1$ . Both species are described by Beverton–Holt type equations [2] showing ideal contest intraspecific competition. The probability of reproduction $g_{i}=f_{i}/(\lambda_{i}N_{i})$ is a function of $N_{i}+b_{ij}N_{j}$ , decreasing most slowly with $N_{i}+b_{ij}N_{j}$ .

Next, the minimum inequality within species 1 can be realized by the limit $s_{1}\to \infty$ ( $u_{1}\to 0$ ). When taking this limit, $b_{12}$ and $s_{1}(e^{u_{1}/\bar{R}}-1)$ must be kept constant as in §3.2. Here, $b_{12}$ can be maintained constant by varying $\alpha_{2}/\alpha_{1}$ simultaneously with $s_{1}$ and $u_{1}$ . In this limit, the competition model (2.9) becomes:


(3.9*a*) $$f_{1}\left(N_{1},N_{2}\right)=\lambda_{1}N_{1}exp\left[-\frac{w_{1}}{\bar{R}}\left(N_{1}+b_{12}N_{2}\right)\right],$$

(3.9*b*) $$\begin{array}{ll} f_{2}(N_{1},N_{2}) & =\lambda_{2}N_{2}{\left[1+(e^{u_{2}/\bar{R}}-1)(N_{2}+b_{21}N_{1})\right]}^{-s_{2}}, \end{array}$$

where $w_{1}=s_{1}u_{1}$ , the actual amount of resources required for a species 1 individual to reproduce, and


$$b_{12}={b_{21}}^{-1}=(1-e^{-u_{2}/\bar{R}})\frac{\bar{R}\alpha_{2}}{u_{1}\alpha_{1}}.$$

Note that in deriving equation (3.9*a*), the following formula for any real number c was used:


$$\lim_{s\to \infty }[1+c/s{]}^{-s}=e^{-c}.$$

The inequality within species 1 disappears completely, and all individuals of species 1 consume resources at the same rate. Equation (3.9*a*) shows that species 1 is described by a Ricker-type equation [1], which is usually used for ideal scramble intraspecific competition. The probability of reproduction of species 1, $g_{1}=f_{1}/(\lambda_{1}N_{1})$ , decreases rapidly as $N_{1}+b_{12}N_{2}$ increases. When $s_{2}=1$ , this model describes interspecific competition between ideal scramblers and ideal contesters. The dynamics of the deterministic version of the same model are discussed in Franke and Yakubu [30].

Finally, consider the case where the inequalities within both species disappear. This can be realized by taking the limit $s_{1},s_{2}\to \infty$ while maintaining $s_{1}u_{1}=w_{1}$ , $s_{2}u_{2}=w_{2}$ and $b_{12}$ constant. Applying this limit to equation (2.9) gives:


(3.10*a*) $$\begin{array}{ll} f_{1}(N_{1},N_{2}) & =\lambda_{1}N_{1}\exp\;\left[-\frac{w_{1}}{\bar{R}}(N_{1}+b_{12}N_{2})\right], \end{array}$$

(3.10*b*) $$\begin{array}{ll} f_{2}(N_{1},N_{2}) & =\lambda_{2}N_{2}\exp\;\left[-\frac{w_{2}}{\bar{R}}(N_{2}+b_{21}N_{1})\right], \end{array}$$

where


$$\begin{array}{c} b_{12}={b_{21}}^{-1}=\frac{u_{2}\alpha_{2}}{u_{1}\alpha_{1}}. \end{array}$$

As the two species have no intraspecific inequality, all conspecifics acquire resources at the same rate, and $b_{ij}$ represents the ratio of the resource acquisition rates of the two species. Owing to the unique case where all conspecifics acquire resources synchronously, a simple interpretation of the reproductive probability $g_{i}=f_{i}/(\lambda_{i}N_{i})$ is possible. For species 1 individuals to reproduce, R must satisfy


$$R\geq w_{1}N_{1}+w_{1}b_{12}N_{2},$$

where $w_{1}b_{12}N_{2}$ on the right-hand side represents the resource consumed by species 2 before each individual of species 1 acquires $w_{1}$ . If this condition is satisfied, all individuals of species 1 reproduce; otherwise none reproduces. As R is exponentially distributed, the reproductive probability $g_{1}=f_{1}/(\lambda_{1}N_{1})$ from equation (3.10*a*) represents the probability of meeting this condition.

## 4. Discussion

Understanding the links between interindividual and interpopulation interactions is ecologically important. This study showed that from a model of random resource competition between individuals of two species, the expected population sizes at the next generation are described by the same functions as in the Hassell–Comins model, whose exponents are related to inequalities in resource allocation between individuals. Considering the exponential distribution of resource R as the most natural (unbiased) probability distribution when only the expected value of R is known [27], the derived Hassell–Comins model (2.9) provides the most natural estimate of the expected population sizes at the next generation in this situation. The functional forms of the original Hassell–Comins model were assumed phenomenologically, rather than derived from interactions between individuals [3,4]. Although the derived equations give expected rather than definite population sizes in the next generation, they have implications for the interpretation of the original Hassell–Comins model, particularly its phenomenological parameters. While inequalities in resource allocation play a significant role, the assumption of thresholds for the amount of resources required for reproduction is equally important. Even if a species as a whole acquires the same amount of resources, the number of individuals acquiring resources above or equal to the threshold should vary with the degree of inequality and so the number of reproductive individuals. However, the inequality within the other species does not affect the reproductive potential, as the impact of the other species stems from the total amount of resources it consumes.

Although there have been previous examples of first-principles derivations of the Hassell–Comins model, this is the first time the model has been derived from an approach related to resource inequality. In Anazawa [26], the Hassell–Comins model was derived in a site-based framework [31,32]. The assumed situation was as follows. Larvae of two species, hatching from eggs laid randomly at numerous resource sites, compete for resources within each site. Only those individuals that have acquired sufficient resources leave the site and lay eggs randomly at sites with renewed resources. Resource inequality was not considered in the competition at each site. Rather, the effect of a clumped distribution of eggs over the sites was considered. When the eggs followed a negative binomial distribution, the derived Hassell–Comins model had exponents related to the degree of clumping. As this derivation did not consider resource inequality, the scenario differed from what Hassell had originally assumed [3]. However, this shows that behind the same phenomenological model there can be different situations. The assumed situation was as follows. Larvae of two species, hatching from eggs laid randomly at numerous resource sites, compete for resources within each site. Only those individuals that have acquired sufficient resources leave the site and lay eggs randomly at sites with renewed resources. Resource inequality was not considered in the competition at each site. Rather, the effect of a clumped distribution of eggs over the sites was considered. When the eggs followed a negative binomial distribution, the derived Hassell–Comins model had exponents related to the degree of clumping. As this derivation did not consider resource inequality, the scenario differed from what Hassell had originally assumed [3]. However, this shows that behind the same phenomenological model, there can be different situations.

Extending the derivation of the Hassell model in Anazawa [27] to two species, this study has obtained an interspecific competition model consistent with the original Hassell–Comins model, which was introduced in an intuitive way from the Hassell model. It might seem that only an expected result has been achieved, but this is not the case. In general, many interspecific competition models are introduced intuitively from single-species population models by replacing the original population size with a linear combination of the population sizes of two species. However, it is not clear under what conditions such an intuitive operation is generally justified. This study obtained results consistent with such an operation, but which may be due to specific assumptions made (e.g. the exponential distribution of resources). The derived model (2.9) is relatively simple but is obtained only after much more detailed consideration of resource competition than in the single-species case. It should also be emphasized that the population-level competition coefficients were obtained as functions of the individual-level parameters and that specific interpretations of these functions were provided. Although this paper considered competition between two species, the discussion in §2.3 can be extended straightforwardly to three or more species. The expected population sizes in the next generation are also described by functions obtained from a simple extension of equation (2.9).

However, the derived model (2.9) faces challenges in being applied repeatedly over generations. This is because equation (2.9) calculates the expected numbers of individuals at the next generation, around which the actual numbers are scattered. Therefore, substituting the expected population sizes at generation t+1 calculated from equation (2.9) into $N_{1}$ and $N_{2}$ on the right-hand sides to determine the expected population sizes at generation t+2 cannot be justified. In general, repeated use of deterministic discrete-time population models will only be possible when population sizes are extremely large and deviations from expected values are negligible. For the model (2.9), both finite population sizes and averaging with the distribution of R make the repeated use difficult. However, the Hassell and Hassell–Comins models have also been used to understand intra- and interspecific competition based on relationships between population sizes in two adjacent generations of experimental populations. It is possible to use the model (2.9) in this way.

This study made rather idealized assumptions about the resource distribution and the pattern of resource acquisition by individuals. The exponential distribution of R is crucial to obtaining analytical results and is consistent with the principle of maximum entropy, but it may not always reflect distributions in reality. Investigating how realistic distributions of R change the results, although difficult to investigate analytically, is a future task. For the resource acquisition, each individual was assumed to acquire only a fixed amount of resources at a time. This idealized assumption was made in order to examine the effects of individual variability in allocated resources in the simplest setting, but in reality, individual variability can arise from a variety of complex factors. For example, it may be more natural to assume that individuals acquire more resources in proportion to the amount of resources they have acquired so far, in which case resource inequality between individuals would be much greater [33]. Further research is needed to investigate how the expected population sizes in the next generation change in such a case.

As it is based on idealized assumptions, equation (2.9) would be difficult to apply directly to understanding real population dynamics and interspecific competition. However, the results may, conversely, suggest that the interactions between individuals captured in the phenomenological Hassell–Comins model are those in such an idealized situation. When studying population dynamics with a realistic individual-based model, the model will be more complex as it requires many detailed assumptions about the interactions between individuals. However, as the Hassell–Comins model (2.9) is expressed in simple formulae, it will be helpful in comparing the results of complex realistic models.

This paper has shown that the expected population sizes at the next generation of two competing species are described by the Hassell–Comins model through stochastic calculations assuming random competition for resources between individuals. The relationship between population- and individual-level parameters, and the effects of inequalities between individuals on the model were also discussed. The findings of this paper will be useful in better understanding the relationship between competition between populations and individual interactions.

## Data Availability

This article does not contain any additional data. It is replicable only by mathematical calculations.

## Appendix A: Derivation of equation (2.8)

This appendix explains the derivation of equation (2.8*a*) from equations (2.6) and (2.7). As geometric distributions appear several times in the appendices, a brief comment on them is made first. Consider a random variable that can take an integer value greater than or equal to 0 and that follows a geometric distribution. Using its mean value $\bar{x}$ , the probability of taking value x can be written as:

(A 1)
$$p(x)=\frac{1}{\bar{x}}{\left(1+\frac{1}{\bar{x}}\right)}^{-x-1}.$$

Starting from equation (2.7),

(A 2)
$${\hat{p}}_{1}(m_{1};\bar{R})=\sum_{M=0}^{\infty }p_{1}(m_{1};M)\;P(M;\bar{R}),$$

we first determine ${\hat{p}}_{1}(m_{1};\bar{R})$ specifically. From equation (2.5), $P(M,\bar{R})$ on the right-hand side of this equation can be written as:

$$P(M,\bar{R})=(1-r)\;r^{M},$$

where $r=e^{-u/\bar{R}}$ , and the mean of M is $\bar{M}=r/(1-r)=(e^{u/\bar{R}}-1{)}^{-1}$ . As $p_{1}(m_{1};M)$ is given by equation (2.2) for $m_{1}\leq M$ and 0 otherwise, equation (A 2) can be rewritten as:

(A 3)
$${\hat{p}}_{1}(m_{1};\bar{R})=\sum_{M=m_{1}}^{\infty }\rho_{1}^{m_{1}}(1-\rho_{1}{)}^{M-m_{1}}\frac{M!}{m_{1}!(M-m_{1})!}\cdot (1-r)\;r^{M}.$$

Relabelling the index M as $M=m_{1}+n$ gives

(A 4)
$${\hat{p}}_{1}(m_{1};\bar{R})=(1-r)(r\rho_{1}{)}^{m_{1}}\sum_{n=0}^{\infty }[r(1-\rho_{1}){]}^{n}\frac{(m_{1}+n)!}{m_{1}!\;n!}.$$

Applying the expansion formula

(A 5)
$$(1-z{)}^{-\nu -1}=\sum_{n=0}^{\infty }z^{n}\frac{(\nu +n)!}{\nu !\;n!},$$

which holds for |z|<1 , to the right-hand side of equation (A 4) with $\nu =m_{1}$ and $z=r(1-\rho_{1})$ , we have:

$$\begin{array}{rl} {\hat{p}}_{1}(m_{1};\bar{R}) & =(1-r)(r\rho_{1}{)}^{m_{1}}[1-r(1-\rho_{1}){]}^{-m_{1}-1}\\ & =\frac{1-r}{r}\frac{1}{\rho_{1}}{\left(1+\frac{1-r}{r}\frac{1}{\rho_{1}}\right)}^{-m_{1}-1}. \end{array}$$

Comparing this equation with equation (A 1) shows that ${\hat{p}}_{1}(m_{1};\bar{R})$ is the geometric distribution:

(A 6)
$${\hat{p}}_{1}(m_{1};\bar{R})=\frac{1}{{\bar{m}}_{1}}{\left(1+\frac{1}{{\bar{m}}_{1}}\right)}^{-m_{1}-1},$$

where

(A 7)
$${\bar{m}}_{1}=\frac{r}{1-r}\rho_{1}=\bar{M}\rho_{1}.$$

From equation (2.6), $f_{1}(N_{1},N_{2})$ is written as:

$$\begin{array}{c} f_{1}(N_{1},N_{2})=\lambda_{1}N_{1}\sum_{m_{1}=s_{1}}^{\infty }{\hat{p}}_{1}(m_{1};\bar{R}), \end{array}$$

and substituting equation (A 6) into the right-hand side of this equation and calculating the infinite sum yields:

(A 8)
$$f_{1}(N_{1},N_{2})=\lambda_{1}N_{1}{\left(1+\frac{1}{{\bar{m}}_{1}}\right)}^{-s_{1}}.$$

Combining equation (A 7) and (2.3) gives:

$$\begin{array}{c} {\bar{m}}_{1}^{-1}=(e^{u/\bar{R}}-1)(N_{1}+\frac{\alpha_{2}}{\alpha_{1}}N_{2}), \end{array}$$

showing that equation (A 8) is identical to equation (2.8*a*).

## Appendix B: Derivation of equation (2.9)

Following the discussion from equation (2.12) onward in §2.3, this appendix explains the derivation of equation (2.9*a*). Substituting equation (2.12) into the right-hand side of equation (2.13), we have:

$$P_{1}(M_{1};\bar{R})=(1-A-B)A^{M_{1}}\sum_{M_{2}=0}^{\infty }B^{M_{2}}\frac{(M_{1}+M_{2})!}{M_{1}!\;M_{2}!},$$

where $A=e^{-u_{1}/\bar{R}}q_{1}$ and $B=e^{-u_{2}/\bar{R}}q_{2}$ . Applying the expansion formula (A 5) to the right-hand side of this equation with $n=M_{2}$ , $\nu =M_{1}$ and z=B , we have:

$$\begin{array}{rl} P_{1}(M_{1};\bar{R}) & =(1-A-B)A^{M_{1}}(1-B{)}^{-M_{1}-1}\\ & =\frac{1-A-B}{A}{\left(\frac{1-B}{A}\right)}^{-M_{1}-1}. \end{array}$$

Comparing this equation with equation (A 1) shows that $P_{1}(M_{1};\bar{R})$ is the geometric distribution:

$$\begin{array}{c} P_{1}(M_{1};\bar{R})=\frac{1}{{\bar{M}}_{1}}{\left(1+\frac{1}{{\bar{M}}_{1}}\right)}^{-M_{1}-1}, \end{array}$$

where

(B 1)
$${\bar{M}}_{1}=\frac{e^{-u_{1}/\bar{R}}\alpha_{1}N_{1}}{(1-e^{-u_{1}/\bar{R}})\alpha_{1}N_{1}+(1-e^{-u_{2}/\bar{R}})\alpha_{2}N_{2}}.$$

With $P_{1}(M_{1};\bar{R})$ obtained here, ${\hat{p}}_{1}(m_{1};\bar{R})$ can be determined from equation (2.14),

(B 2)
$${\hat{p}}_{1}(m_{1};\bar{R})=\sum_{M_{1}=0}^{\infty }p_{1}(m_{1};M_{1})\;P_{1}(M_{1};\bar{R}),$$

where

(B 3)
$$p_{1}(m_{1};M_{1})={\left(\frac{1}{N_{1}}\right)}^{m_{1}}{\left(1-\frac{1}{N_{1}}\right)}^{M_{1}-m_{1}}\frac{M_{1}!}{m_{1}!(M_{1}-m_{1})!}$$

is the distribution of $m_{1}$ when $M_{1}$ is fixed. A comparison of the pair of equations (B 2) and (B 3) with equation (A 3) shows that the infinite sum on the right-hand side of equation (B 2) is equivalent to that of equation (A 3) with the replacements $\rho_{1}\to 1/N_{1}$ , $M\to M_{1}$ and $\bar{M}\to {\bar{M}}_{1}$ . Therefore, ${\hat{p}}_{1}(m_{1};\bar{R})$ here is expressed as:

$${\hat{p}}_{1}(m_{1};\bar{R})=\frac{1}{{\bar{m}}_{1}}{\left(1+\frac{1}{{\bar{m}}_{1}}\right)}^{-m_{1}-1}$$

as in equation (A 8), where ${\bar{m}}_{1}$ is replaced by:

(B 4)
$${\bar{m}}_{1}={\bar{M}}_{1}/N_{1}.$$

Similarly, $f_{1}(N_{1},N_{2})$ is given by:

(B 5)
$$f_{1}(N_{1},N_{2})=\lambda_{1}N_{1}{\left(1+\frac{1}{{\bar{m}}_{1}}\right)}^{-s_{1}}$$

as in equation (A 8). Combining equations (B 4) and (B 1) yields:

$${\bar{m}}_{1}^{-1}=e^{u_{1}/\bar{R}}\left[(1-e^{-u_{1}/\bar{R}})N_{1}+(1-e^{-u_{2}/\bar{R}})\frac{\alpha_{2}}{\alpha_{1}}N_{2}\right],$$

showing that equation (B 5) is equivalent to equation (2.9*a*).
